# Multi-trait and multi-environment genomic prediction for flowering traits in maize: a deep learning approach

**DOI:** 10.3389/fpls.2023.1153040

**Published:** 2023-08-01

**Authors:** Freddy Mora-Poblete, Carlos Maldonado, Luma Henrique, Renan Uhdre, Carlos Alberto Scapim, Claudete Aparecida Mangolim

**Affiliations:** ^1^ Institute of Biological Sciences, University of Talca, Talca, Chile; ^2^ Centro de Genómica y Bioinformática, Facultad de Ciencias, Universidad Mayor, Santiago, Chile; ^3^ Department of Agronomy, State University of Maringá, Paraná, Brazil; ^4^ Department of Biotechnology, Genetics and Cell Biology, State University of Maringá, Paraná, Brazil

**Keywords:** Bayesian models, deep learning, multi-trait, multi-environment, genomic prediction, candidate genes

## Abstract

Maize (*Zea mays* L.), the third most widely cultivated cereal crop in the world, plays a critical role in global food security. To improve the efficiency of selecting superior genotypes in breeding programs, researchers have aimed to identify key genomic regions that impact agronomic traits. In this study, the performance of multi-trait, multi-environment deep learning models was compared to that of Bayesian models (Markov Chain Monte Carlo generalized linear mixed models (MCMCglmm), Bayesian Genomic Genotype-Environment Interaction (BGGE), and Bayesian Multi-Trait and Multi-Environment (BMTME)) in terms of the prediction accuracy of flowering-related traits (Anthesis-Silking Interval: ASI, Female Flowering: FF, and Male Flowering: MF). A tropical maize panel of 258 inbred lines from Brazil was evaluated in three sites (Cambira-2018, Sabaudia-2018, and Iguatemi-2020 and 2021) using approximately 290,000 single nucleotide polymorphisms (SNPs). The results demonstrated a 14.4% increase in prediction accuracy when employing multi-trait models compared to the use of a single trait in a single environment approach. The accuracy of predictions also improved by 6.4% when using a single trait in a multi-environment scheme compared to using multi-trait analysis. Additionally, deep learning models consistently outperformed Bayesian models in both single and multiple trait and environment approaches. A complementary genome-wide association study identified associations with 26 candidate genes related to flowering time traits, and 31 marker-trait associations were identified, accounting for 37%, 37%, and 22% of the phenotypic variation of ASI, FF and MF, respectively. In conclusion, our findings suggest that deep learning models have the potential to significantly improve the accuracy of predictions, regardless of the approach used and provide support for the efficacy of this method in genomic selection for flowering-related traits in tropical maize.

## 1 Introduction

Maize (*Zea mays* L.) is a crucial cereal crop that plays a vital role in global food security, biofuel production, and animal feed (Maldonado et al., 2020; Grote et al., 2021). Consumed by over 4.5 billion people, particularly in rural areas of Latin America and Africa, it is an important source of calories and nutrients (Domínguez-Hernández et al., 2022). With its high genetic diversity and ease of sexual reproduction, maize is a versatile crop that offers many agronomic and reproductive advantages. Its separate inflorescences (male and female) also allow for easily controlled crosses and the creation of highly inbred lines – high levels of genetic homozygosity in the lines – (Strable and Scanlon, 2009). As a result of these advantages, variousss agronomic traits such as grain yield, flowering time, and nutritional value have been improved through breeding programs worldwide (e.g., Alves et al., 2018; Gedil and Menkir, 2019).

Flowering time is an agriculturally important trait for crop production that can be manipulated by various approaches such as breeding and genetic modifications (Hirohata et al., 2022). In maize, it has been shown that flowering time is significantly associated with regional adaptation and is a complex trait controlled by hundreds of loci with small effects, many with multiple allelic series (Romero et al., 2017). The genetic control of flowering time involves networks of genes that interact with environmental conditions, which is a determining factor in the duration of the crop cycle (Parent et al., 2018). Conventional approaches in quantitative genetics, such as QTL (Quantitative Trait *loci*) mapping, genomic selection, and genome-wide association studies (GWAS), have traditionally been used to investigate the genetic basis of the quantitative variation in flowering time-related traits. For example, Romero et al. (2017) assayed the potential for predicting flowering time in maize landraces using GBLUP (Genomic Best Linear Unbiased Predictor); a widely used statistical method for genomic selection. Across trials, the average fivefold cross-validated prediction accuracy was 0.45 for flowering time using either 30,000 markers or one SNP for each of the most significant genes. Similarly, Maldonado et al. (2020) used deep learning predictive models and found a predictive ability of up to 0.78 for maize traits related to flowering.

Other genetic studies in maize have emphasized the importance of identifying genetic variants (QTLs) controlling flowering time-related traits under a wide range of environmental conditions to improve stress tolerance (Leng et al., 2022). The study conducted by Maldonado et al. (2019) who used a population of inbred lines of tropical maize, identified a total of 45 SNPs and 44 Haplotype-block significantly associated with flowering time, which was distributed across the entire genome. Moreover, the study also found that some of the loci identified were associated with multiple flowering-related traits, which suggests a possible pleiotropic effect of these loci. Additionally, the study found that some loci displayed associations with multiple flowering-related traits. This observation suggests the presence of a potential pleiotropic effect, where a single genetic locus influences the expression of multiple traits related to flowering. Another study carried out by Birnbaum and Roberts (2019), which aimed to identify SNPs significantly associated with flowering time in a panel of maize inbred lines by using a GWAS approach, identified a total of 25 significant SNPs for flowering time, of which 15 were novel, and 10 were previously reported. The study also identified several candidate genes underlying the significant SNPs that were associated with flowering traits. Overall, these studies demonstrate that GWAS can provide valuable information for understanding the genetic basis of flowering time-related traits, which can inform the development of improved maize varieties.

Various studies have highlighted the potential of using GWAS and genomic selection approaches in enhancing crop breeding and developing improved maize varieties (e.g., Liu et al., 2021; Ma and Cao, 2021; Vinayan et al., 2021). For example, the study conducted by Zhou et al. (2021) aimed to identify the genetic variants associated with yield and yield-related traits in maize crops. The results of the study found that the combination of these methods provided the best results in predicting the breeding value of individuals for yield and yield-related traits. Furthermore, the study identified several loci associated with yield and yield-related traits, which demonstrate the effectiveness of the combined GWAS and genomic selection approach in identifying genetic variants associated with these traits. On the other hand, recent studies have placed significant emphasis on the advancement of more precise predictive models, such as multi-trait or multi-environment genomic prediction models. These models have shown remarkable improvements in prediction accuracy when compared to uni-trait models, especially when traits are correlated. Additionally, they have proven beneficial in predicting traits that are difficult or expensive to phenotype (Gill et al., 2021). As breeders routinely gather phenotypic data across numerous traits and diverse environments, extending the application of multi-trait approaches to incorporate genotype-by-environment interactions could further enhance the accuracy of genomic prediction models within breeding programs (Montesinos-López et al., 2019; Hu et al., 2022). Multi-trait and multi-environment Bayesian and Deep Learning models have been proposed by Montesinos-López et al. (2016) (Bayesian multi-trait and multi-environment; BMTME), Montesinos-López et al. (2018) (Deep learning multi-trait and multi-environment; DL), Granato et al., 2018 (Bayesian Genomic Genotype × Environment Interaction; BGGE) and Hadfield and Nakagawa (2010) (MCMC Generalised Linear Mixed Models; MCMCglmm). Sandhu et al. (2022) showed that the multi-trait DL approach improved the accuracy of genomic prediction compared to uni-trait and multi-trait+multi-environment (BMTME) models. This highlights the potential of using multi-trait, multi-environment deep learning models in genomic prediction and crop breeding. The study highlights the potential of using multi-trait, multi-environment deep learning models in genomic prediction and crop breeding.

Uni- and Multi-trait (UT and MT, respectively), as well as, Uni- and Multi-environment (UE and ME, respectively) approaches have been compared keeping fixed the traits (UTUE vs UTME, or MTUE vs MTME) or environments (UTUE vs MTUE, or UTME vs MTME) as one (Uni) or multiple (Multi). However, comparisons among all approaches simultaneously have not been performed yet, particularly for traits exhibiting low or negative correlations. Thus, the present study aimed to evaluate the performance of these four approaches for the genomic prediction of flowering-related traits in tropical maize using the Bayesian and deep learning approaches. To accomplish this, a panel of 258 tropical maize inbred lines was analyzed using SNP markers. In addition, a complementary genome-wide association study, coupled with network-assisted gene prioritization (post-GWAS), was performed to identify potential candidate genes associated with these traits. The results of this study provide insights into the potential of using deep learning models for enhancing prediction accuracy in the context of genomic selection for flowering-related traits in tropical maize.

## 2 Materials and methods

### 2.1 Plant materials

The study utilized a panel of 258 tropical maize inbred lines from the core collection germplasm of the State University of Maringa, Parana State, Brazil, which were derived from three genetic backgrounds: field corn, popcorn, and sweet corn genotypes (**Supplementary Table** **S1**). Genomic prediction models were developed using phenotypic records derived from three locations within the state of Paraná, Brazil: Cambira, Sabaudia and Iguatemi, during the growing seasons of 2017-2018 (Cambira and Sabaudia), 2019-2020 (Iguatemi), and 2020-2021 (Iguatemi). Complementary, a genome-wide association study was performed using Iguatemi data (both growing seasons), and then, these results were compared with the other locations following the study by Maldonado et al. (2019).

### 2.2 Experimental design and trait measurement

The experimental design for Cambira and Sabaudia was an alpha-lattice with 24 incomplete blocks and 3 replications per line, while in Iguatemi, the lines were planted according to a partially balanced incomplete block design in a 17x17 square lattice with 4 replications per line. The following flowering-related traits were evaluated: Female Flowering time (FF) measured as the number of days from sowing to visible silks, Male Flowering time (MF) measured as the number of days from sowing to anther extrusion from the tassel glumes, and Anthesis-Silking Interval (ASI) calculated as the difference between MF and FF (Maldonado et al., 2019; Maldonado et al., 2020).

### 2.3 Phenotypic data analysis

The analysis of the phenotypic data was performed using the following Bayesian model available in the package “MCMCglmm” (Hadfield and Nakagawa, 2010) of R software (Team R. C, 2013):


(1) y=Xβ+Zf+ϵ

where y is the vector of the phenotypic observations, X and Z are the known incidence matrices that relate the observation vector (y) to the vectors β and f, respectively. β is the vector of replications and block within replications, f is the vector of family effects and ϵ is the vector of residuals or error vector. The *y* vector corresponds to the adjusted phenotypic observations, which were utilized in the subsequent sections for Genomic Prediction Models and Genome-Wide Association Study.

Correlations between each pair of traits were calculated using a Bayesian bi-trait model (MCMCglmm), according to Maldonado et al. (2019), using the following expression:


(2) $$r_{xy}=\frac{{\hat{\sigma }}_{G_{xy}}}{\sqrt{{\hat{\sigma }}_{G_{x}}^{2}*{\hat{\sigma }}_{G_{y}}^{2}}}$$

where ${\hat{\sigma }}_{G_{xy}}$ correspond to posterior distribution samples of genotypic covariance between the traits, and ${\hat{\sigma }}_{G_{x}}^{2}$ , ${\hat{\sigma }}_{G_{y}}^{2}$ correspond to posterior mean distribution samples of genotypic variance for each pair of traits under analysis.

### 2.4 Genotyping, population structure and linkage disequilibrium

Genomic DNA was extracted from the leaf tissue of 21-day-old plants using the protocol described by Maldonado et al. (2019), which follows the method developed by Chen and Ronald (1999). The DNA samples were then sent to the University of Wisconsin-Madison Biotechnology Center for SNP discovery through genotyping by sequencing (Elshire et al., 2011; Glaubitz et al., 2014). Monomorphic SNP markers and those with a call rate lower than 90% were removed, and SNPs with a minor allele frequency (MAF) of less than 0.05 were eliminated, resulting in 291,633 high-quality SNPs. Finally, missing data were imputed through linkage disequilibrium k-nearest neighbor imputation (Money et al., 2015), as described in Maldonado et al. (2020).

The kinship matrix was calculated using the identity-by-state method (Endelman and Jannink, 2012) with the TASSEL 5.2 software (Bradbury et al., 2007). The population genetic structure was inferred using a Bayesian clustering model in the InStruct 2.3.4 program (Gao et al., 2007). Ten runs were performed for each possible value of K (number of genetically differentiated groups), ranging from 1 to 6, with 100,000 Monte Carlo Markov Chain replicates and a burn-in period of 10,000 iterations. The optimal value of K was determined using the second-order change rate of the probability function with respect to K (ΔK), as proposed by Evanno et al. (2005) and the lowest deviance information criterion (DIC). Additionally, a t-distributed stochastic neighbor embedding (t-SNE) visualization was performed using Python 3.7 language and the Keras 2.2.4 and TensorFlow 1.14.0 libraries to corroborate the results from InStruct. A perplexity of 30, a learning rate of 200 and 1,000 iterations were used in the t-SNE model according to López-Cortés et al. (2020).

The Linkage Disequilibrium (LD) was estimated using the correlation coefficients of allelic frequencies (r2) calculated for all possible allele combinations. The critical r2 value was determined using the transformation of the square root of the r2 values as proposed by Breseghello and Sorrells (2006), with the 95th percentile of these data serving as the threshold.

### 2.5 Genomic prediction models and cross-validation

#### 2.5.1 Markov chain Monte Carlo generalized linear mixed models

In this study, Uni-Trait-Uni-Environment and Multi-Trait-Uni-Environment analyses were implemented according to Mathew et al. (2016) and Torres et al. (2018). The Uni- and Multi-Trait approaches were implemented using the following model:


(3) $$y_{i}=X_{i}\beta_{i}+Z_{i}u_{i}+\epsilon_{i},\;\;\;\;i=1,\;2\ldots ,\;n$$

where *y_i_* is the vector of the phenotypic values of the traits, *β_i_* and *u_i_* are vectors of fixed and random effects associated with trait *i*, respectively, and *ϵi* is a vector of error terms, which are independently normally distributed with mean zero and variance $\sigma_{e}^{2}$ . Moreover, *X_i_* and *Z_i_* are incidence matrices for the fixed and random effects for trait *i*, respectively. Then mixed model equation (MME) for the above model is:


(4) $$\left[\begin{array}{cc} X^{\prime }R^{-1}X & X^{\prime }R^{-1}Z\\ Z^{\prime }R^{-1}X & Z^{\prime }R^{-1}Z+G^{-1} \end{array}\right]\left[\begin{array}{c} \beta \\ u \end{array}\right]=\left[\begin{array}{c} X^{\prime }R^{-1}y\\ Z^{\prime }R^{-1}y \end{array}\right]$$

where *R* and *G* are covariance matrices associated with the vectors *ϵ* and *u* of residuals and random effects, respectively. If *R0* is the residual covariance for more than one trait, then *R* can be calculated as *R=R0*⊗*I* (⊗ represent the Kronecker product between *R0* and the identity matrix). Similarly, the genetic covariance matrix *G* can be calculated as *G=G0*⊗*A*, where *A* and *G0* are the additive genetic relationship matrix and additive genetic (co)variance matrix, respectively. The MCMCglmm R package (Hadfield and Nakagawa, 2010; Team R. C, 2013) was used to implement the model, using 100,000 iterations, a 10,000 burn-in period, and a sampling interval of 5.

#### 2.5.2 Bayesian genomic genotype × environment interaction

The Uni-Trait-Multi-Environment approach was implemented using the BGGE R package (Granato et al., 2018) within R software (Team R. C, 2013). This package utilizes Bayesian hierarchical modeling to solve linear mixed models, as described in Granato et al. (2018) and Costa-Neto et al. (2020), in which the distribution of the transformed data *d*⁠, given *b* and $\sigma_{\epsilon }^{2}$ , is:


(5) $$f\left(d|b,\sigma_{\epsilon }^{2}\right)=\prod_{i=1}^{n}N\left(d_{i}|b_{i},\sigma_{\epsilon }^{2}\right)$$

The Bayesian linear mixed model assumes that $p\left(u|\sigma_{u}^{2}\right)=N\left(u|0,K\sigma_{u}^{2}\right)$ ; the conditional distribution of *b_i_* is given as $p\left(b_{i}|\sigma_{u}^{2}\right)=N\left(b_{i}|0,K\sigma_{u}^{2}s_{i}\right)$ , where si is the eigenvalues. The BGGE package assumes that conjugate prior distribution of $\sigma_{u}^{2}$ and $\sigma_{\epsilon }^{2}$ are given by inverse chi-squared with $p\left(\sigma_{\epsilon }^{2}\right)\sim \chi^{-2}\left(v_{u},Sc_{u}\right)$ and $p\left(\sigma_{\epsilon }^{2}\right)\sim \chi^{-2}\left(v_{\epsilon },Sc_{\epsilon }\right)$ , respectively, in which $v_{u}$ and $v_{\epsilon }$ denote the degree of freedom, and $Sc_{u}$ and $Sc_{\epsilon }$ the scale factors for µ and ϵ. Then, the joint posterior distribution of (*b*, $\sigma_{u}^{2}$ , $\sigma_{\epsilon }^{2}$ ), given d, $v_{\mu }$ , $Sc_{u}$ , $v_{\epsilon }$ , $Sc_{\epsilon }$ and *S*⁠, is:


(6) $$\begin{array}{l} p\left(b,\sigma_{u}^{2},\sigma_{\epsilon }^{2}|d,v_{u},Sc_{u},v_{\epsilon },Sc_{\epsilon },S\right)\propto \left\{\prod_{i=1}^{n}N\left(d_{i}|b_{i},\sigma_{\epsilon }^{2}\right)\;N\left(b_{i}|0,\sigma_{u}^{2}s_{i}\right)\right\}\times \chi^{-2}\left(\sigma_{u}^{2}|v_{u},v_{u}Sc_{u}\right)\\ \times \chi^{-2}\left(\sigma_{\epsilon }^{2}|v_{\epsilon },v_{\epsilon }Sc_{\epsilon }\right) \end{array}$$

Finally, the BGGE analysis was conducted using 100,000 iterations, with a 10,000 iteration burn-in period and a thinning of 5.

#### 2.5.3 Bayesian multi-trait and multi-environment

The Multi-Trait-Multi-Environment analysis was carried out using the BMTME R package (Montesinos-López et al., 2016) within R software (Team R. C, 2013). The BMTME model is defined as (Montesinos-López et al., 2018; Sandhu et al., 2022):


(7) $$y=X\beta +Z_{1}b_{1}+Z_{2}b_{2}+\epsilon$$

where *y* is the matrix of order *t* x *l*, with *t* is the number of traits and *l* = *e* x *g*, where *e* and *g* are the numbers of environments and genotypes, respectively; *X*, *Z_1_*, and *Z_2_* are design matrixes for environmental effect, genotypic effect, and genotype by environmental interaction, respectively; *β* is beta coefficient matrix of order *e* x *t*; *b_1_* is the random genotypic effect of genotype × trait interaction distributed as *b_1_* ∼ MN(0, *G*, ∑*t*), where *G* is additive relationship matrix calculated using the VanRaden (2008) and ∑*t* is the unstructured covariance matrix of order *t* x *t*; *b_2_* is the random genotypic x trait x environment effect matrix distributed as *b_2_* ∼ MN(0, ∑*e G*, ∑*t*), where ∑*e* is the unstructured covariance matrix of order *e* x *e*. BMTME was performed considering 10,000 burn-in and 100,000 test iterations.

#### 2.5.4 Uni- and multi-trait, uni- and multi-environment deep learning

**Figure 1:**
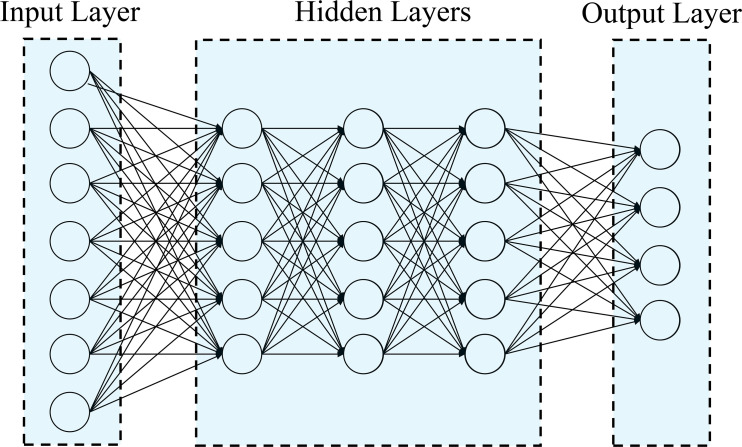
Example of feedforward deep neural network with one input layer (with n neurons that correspond to the input information), three hidden layers (each layer with M neurons) and one output layer (with o neurons that correspond to number of traits to be predicted).

In this study, Deep Learning methods were used to analyze Uni- and Multi-Trait, Uni- and Multi-Environment data, as described in Montesinos-López et al. (2018); Crossa et al. (2019) and Montesinos-López et al. (2019). A densely connected network was chosen as it does not assume a specific structure for the input features. This network typically includes an input layer, T output layers (for multi-trait modeling), and hidden layers between the input and output layers. This type of neural network is commonly referred to as a feedforward neural network (**Figure 1**).

In this study, we employed a neural network architecture with multiple layers to predict flowering traits in tropical maize (**Figure 1**). The network consists of an input layer with “n” neurons, representing the number of features in the dataset. Following the input layer, three hidden layers were incorporated, each containing 50 neurons. These hidden neurons perform non-linear transformations on the original input attributes, as described by Montesinos-López et al. (2018). For the output layer, the network was designed to have one neuron for uni-trait predictions and four neurons for multi-trait predictions. The number of output neurons corresponds to the number of response variables we aimed to predict for flowering traits. The neurons in the network are fully connected, and the strength of the connection weights determines the contribution of each neuron to the overall network output. A regularization technique known as dropout was implemented to temporarily removes a random subset of neurons and their connections during the training process, enhancing the network’s ability to generalize and avoid overfitting (Montesinos-López et al., 2019).

The analytical forms of the model depicted in **Figure 1** can be represented by the following equation (Montesinos-López et al., 2019):


(8) $$V=g\left(\sum_{p=1}^{N}w_{jp}V_{p}\right)\;\text{for hidden layers}$$

(9) $$y_{o}=g\left(\sum_{p=1}^{N}w_{op}V_{p}\right)\;\text{for output layer}$$

where N denotes the total number of input variables in each layer, $w_{jp}$ and $w_{jp}$ represents the weight of the input in hidden (with *j*=1, …, M neurons) and output (with *o*=1, …, O neurons) layers, respectively, while $V_{p}$ represents the value of the *p*th input variable, and *g* represents the activation function. In this network, each layer generates the output for each neuron in the subsequent layer, ultimately producing the output for each response variable of interest. The learning process involves adjusting the weights that connect the layers to optimize the model’s performance. The input variables for the multi-trait approach corresponded to the concatenation of environments, markers through the Cholesky decomposition of the genomic relationship matrix, and genotype × environment interaction (*G*×*E*). For this purpose, the design matrices of environments (*ZE*), genotypes (*ZG*) and *G*×*E* (*ZGE*) were built, followed by the Cholesky decomposition of the genomic relationship matrix (*G*). Then, the design matrix of genotypes was post-multiplied by the transpose of the upper triangular factor of the Cholesky decomposition (*QT*), $Z_{G}^{*}=Z_{G}Q^{T}$ , followed by the calculation of the *G*×*E* term as the product of the design matrix of the *G*×*E* term post-multiplied by the Kronecker product of the identity matrix of order equal to the number of environments and *QT*, that is, $Z_{GE}^{*}=Z_{GE}\left(I_{I}⊗Q^{T}\right)$ . After that, the matrix with input covariates used for implementing Deep Learning models was equal to $X=\left[Z_{E},Z_{G}^{*},Z_{GE}^{*}\right]$ . It should be noted that Uni-Trait approach uses the same implementation as the multi-trait approach described above but with a feedforward neural network with only one neuron in the output layer.

In this study, deep learning models were implemented using the R code of Montesinos-López et al. (2018) in R software (Team R. C, 2013). The following hyperparameters were considered: 50 units (U), 200 epochs, 3 hidden layers, rectified linear activation unit (ReLU) as the activation function, and the dropout regularization method for training the models.

#### 2.5.5 Cross validation

The genomic prediction methods were evaluated using four approaches: Uni-Trait-Uni-Environment (UTUE), Uni-Trait-Multi-Environment (MTUE), Multi-Trait-Uni-Environment (MTUE) and Multi-Trait-Multi-Environment (MTME). These approaches were tested in two scenarios: I) randomly selecting independent training (80%) and validation (20%) groups (for each trait in each site), in which 50 cycles of cross-validation were performed, and II) predicting the second season of environment Iguatemi (validation dataset) using the first season of environment Iguatemi (training dataset DT1), other environments (Cambira and Sabaudia; training dataset DT2), and other environments (Cambira and Sabaudia) plus the first season of Iguatemi (training dataset DT3).

The prediction accuracy was evaluated by calculating the average Pearson correlation coefficient between the observed and predicted phenotypes in the validation set for all models (Deep Learning, MCMCglmm, BMTME, and BGGE).

### 2.6 GWAS, candidate genes and co-functional networks

The Genome-Wide Association Study (GWAS) was conducted using the mixed linear model (MLM) in TASSEL 5.2 (Bradbury et al., 2007) for the three flowering traits (FF, MF, and ASI). The statistical model incorporated the effects of population structure (Q) and genetic relationships or kinship matrix (K) among the inbred lines, as represented by the following mixed model:


(10) y = Sα + Qv + Zμ + ϵ

where *y* is the vector of adjusted phenotypic observations, *α* and *v* are the vectors of fixed effects of molecular markers and population structure, respectively, *μ* and *ϵ* are the vectors of random effects of polygenic effects and residual, respectively. S, Q and Z are the incidence matrices of the associated vectors.

The probability of a locus being associated with two or more traits was evaluated using the Bayes Factor (BF) and Posterior Probability of Association (PPA) (Stephens and Balding, 2009). The PPA was calculated by considering the BF and prior probability of association, as outlined by Stephens and Balding (2009):


(11) $$PPA=\frac{\left(BF\;x\;\pi \right)}{\left(1-\pi \right)+\left(BF\;x\;\pi \right)}$$

where *π* is the significance level of SNP associated with the trait of interest. BF was calculated using Bayesian multivariate regression analysis in the SNPTEST software (Marchini and Band, 2016) according to Maldonado et al. (2019).

The candidate genes surrounding the significant SNPs identified by GWAS were selected by establishing a window of twice the distance indicated by the LD around the SNP, with the SNP serving as the center of the window. These candidate genes were then prioritized using MaizeNet (Lee et al., 2019) by analyzing their connections to genes previously associated with flowering time in *Zea mays*. Co-functional networks were also constructed by linking the candidate genes to subnetworks enriched for gene ontology annotations related to biological processes involved in flowering.

GWAS, identified candidate genes, and constructed co-functional networks were applied for the Iguatemi, seasons 1 and 2. Results for the Cambira and Sabaudia environments can be found in the study by Maldonado et al. (2019).

## 3 Results

**Figure 2:**
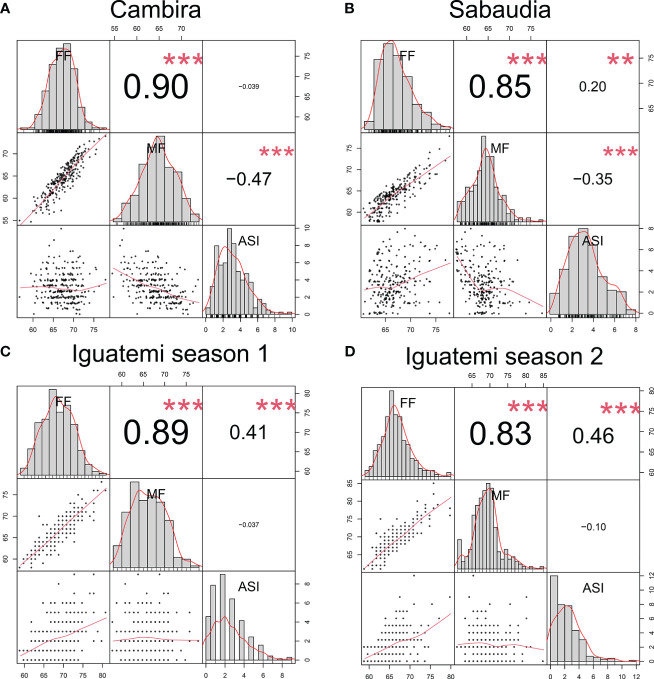
Correlation between flowering traits in the Cambira, Sabaudia, Iguatemi season 1, and Iguatemi season 2 environments (**A–D**, respectively). The figure illustrates the correlation between female flowering (FF), male flowering (FM), and anthesis-silking interval (ASI) in the four different environments. The diagonal of the plot displays histograms and distributions of the observed phenotype values, while the lower off-diagonal presents scatter plots between the traits. Significance levels of the correlation coefficients are indicated by ** for p< 0.01, and *** for p< 0.001.

**Figure 3:**
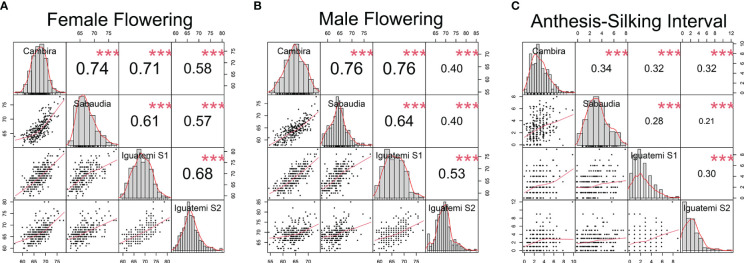
Correlation among the study environments (Cambira, Sabaudia, Iguatemi season 1 and Iguatemi season 2) for each flowering trait: female and male flowering (**A, B**, respectively); and anthesis-silking interval **(C)**. The diagonal line of the plot illustrates the histograms and the distribution of the observed phenotype values for each trait across all environments. The lower off-diagonal section presents the scatterplot between the environments for each trait, whereas the upper off-diagonal section displays the correlation coefficient between environments for each trait. Significance levels of the correlation coefficients is indicated by *** for p< 0.001.

In this study, the genetic correlations between female flowering (FF) and male flowering (MF) remained consistent across all environments (**Figure 2**) with a positive correlation (r > 0.82) and highly significant (p<0.001). However, the correlation between the anthesis-silking interval (ASI) and the other two traits was inconsistent across environments, showing both positive and negative correlation values. Furthermore, the correlation of the flowering traits among the different environments (Cambira, Sabaudia, Iguatemi season 1, and Iguatemi season 2) were positive and statistically significant (**Figure 3**). Notably, MF had the highest correlations among the environments Cambira, Sabaudia and Iguatemi season 1, while FF had the highest correlation values among Iguatemi season 2 and other environments (**Figure 3**).

### 3.1 Genetic structure and linkage disequilibrium

In this study, a Bayesian clustering analysis was conducted on 258 tropical inbred lines, resulting in the grouping of these lines into two genetic clusters (as determined by the lowest DIC value and the highest ΔK). Cluster I and II consisted of 83 (with 82 popcorn and one field corn genotypes) and 175 maize lines (comprising 151 field corn, 13 popcorn, and all sweet corn lines) respectively.

**Figure 4:**
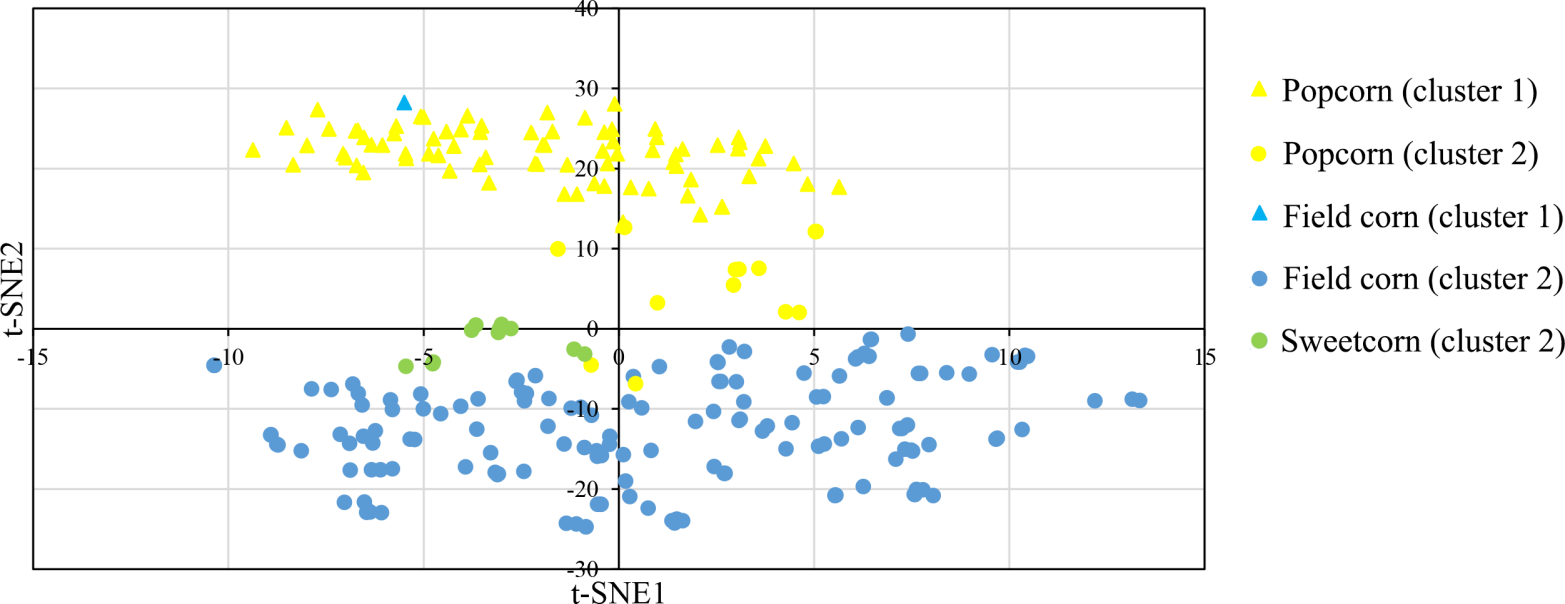
t-distributed stochastic neighbor embedding (t-SNE) visualization of the genetic relatedness of 258 maize inbred lines using a genome-wide panel of 291,633 SNP markers. The visualization is color-coded by population, with yellow representing Popcorn, blue representing Field corn, and green indicating Sweetcorn. The shapes of the individual points indicate an individual’s proportion of ancestry to genetically differentiated groups determined by InStruct, with triangles indicating cluster 1 and circles indicating cluster 2.

The t-SNE method was used to visualize the SNP data, and it clearly separated the two clusters through its second dimension (t-SNE2), which was consistent with the results obtained from InStruct (**Figure 4**). The t-SNE method effectively maintained the distributions of the original data space (by matching pairwise similarity distributions) in a lower-dimensional projected space (Chan et al., 2018).

Linkage disequilibrium (LD) was also estimated at the genome-wide level and for each individual chromosome (**Supplementary T** **able S2**). The LD decayed rapidly within 2.7 kb, with a cut-off value of r2 = 0.12. Chromosomes 3 and 7 showed a faster LD decay than the other chromosomes, with values of about 2.12 kb and a cut-off of r2 = 0.12. Conversely, chromosome 4 presented the slowest LD decay, with a value of 5.35 kb.

### 3.2 Genome-wide association study

The results of the genome-wide association study (GWAS) for the flowering traits in Iguatemi seasons are presented in **Table 1**. A total of 31 SNPs were identified as being associated with the three traits of interest across both Iguatemi seasons, with 13 SNPs associated in the first season (Iguatemi 2020), 18 in the second season (Iguatemi 2021), and one in both seasons. Of these, 11 SNPs were associated with ASI, with 5 identified in the first season and 6 in the second season. Similarly, 11 SNPs were found to be associated with FF, with 5 identified in season 1 and 6 in season 2. Lastly, 9 SNPs were associated with FM, with 3 identified in season 1 and 6 in season 2. Notably, two SNPs (S5_217372319 and S6_150165479) were concomitantly associated with both FF and FM traits, suggesting a possible pleiotropic effect. To confirm this, multivariate Bayesian regression was performed on these loci in relation to the FF and FM traits. This analysis yielded PPA values of 0.99 and 0.74 for S6_150165479 and S5_217372319, respectively, and log10 (BF) > 5.1 for both loci, further supporting the pleiotropic effect of these loci as indicated in the **Supplementary Table** **S3**.

**Table 1:** Summary of the associations detected by a genome-wide association study for the traits of female/male flowering time (FF and MF, respectively) and anthesis–silking interval (ASI).

Trait	Iguatemi Season	Marker	Chr	Pos	p-value	PV%	BIN
ASI	1	S1_214720998	1	214720998	5.53X10^-06^	7.9%	1.07
	1	S1_95747751	1	95747751	5.56X10^-06^	7.8%	1.05
	1	S1_12340947	1	12340947	1.40X10^-05^	7.1%	1.01
	1	S2_142739572	2	142739572	1.46X10^-05^	7.2%	2.05
	1	S8_112412901	8	112412901	1.86X10^-05^	7.1%	8.04
	2	S4_245982321	4	245982321	7.40X10^-07^	9.6%	4.11
	2	S1_214720998	1	214720998	5.43X10^-06^	8.0%	1.07
	2	S3_122398302	3	122398302	7.91X10^-06^	7.8%	3.04
	2	S3_122398313	3	122398313	7.91X10^-06^	7.8%	3.04
	2	S3_122398320	3	122398320	7.91X10^-06^	7.8%	3.04
	2	S4_245029688	4	245029688	9.45X10^-06^	7.6%	4.11
FF	1	S8_18474015	8	18474015	2.65X10^-06^	8.0%	8.02
	1	S6_150165479	6	150165479	5.00X10^-06^	7.5%	6.05
	1	S10_14797601	10	14797601	8.82X10^-06^	7.2%	10.03
	1	S8_143046924	8	143046924	9.44X10^-06^	7.2%	8.05
	1	S7_468747	7	468747	1.74X10^-05^	6.7%	7.00
	2	S5_42052202	5	42052202	3.89X10^-06^	7.8%	5.03
	2	S5_217372319	5	217372319	1.82X10^-05^	7.6%	5.09
	2	S2_15002111	2	15002111	1.11X10^-05^	7.2%	2.02
	2	S2_47411894	2	47411894	1.27X10^-05^	7.0%	2.04
	2	S2_43966599	2	43966599	1.40X10^-05^	6.9%	2.04
	2	S7_34488540	7	34488540	1.40X10^-05^	7.0%	7.02
FM	1	S9_144119131	9	144119131	5.78X10^-06^	7.6%	9.06
	1	S6_150165479	6	150165479	6.58X10^-06^	7.4%	6.05
	1	S7_13731608	7	13731608	7.25X10^-06^	7.3%	7.01
	2	S5_217372319	5	217372319	1.05X10^-06^	8.8%	5.09
	2	S2_222099831	2	222099831	5.57X10^-06^	7.6%	2.08
	2	S1_16300644	1	16300644	9.18X10^-06^	7.2%	1.02
	2	S1_65858162	1	65858162	1.02X10^-05^	7.2%	1.04
	2	S7_8758861	7	8758861	1.16X10^-05^	7.1%	7.01
	2	S3_218088466	3	218088466	1.65X10^-05^	6.8%	3.09

In season 1 (Iguatemi 2020), the proportion of the phenotypic variance (PV%) explained by SNP markers was 37%, 37%, and 22% of the phenotypic variation of ASI, FF, and FM, respectively (**Table 1**). In season 2 (Iguatemi 2021), the PV% explained for ASI and FM was higher than in the first season, at 49% and 45%, respectively.

### 3.3 Candidate genes and co-functional networks

A total of 26 candidate genes were identified based on the physical position of these SNPs in relation to the maize reference genome B73 (**Supplementary Table S4**). These candidate genes were found to be neighboring to the associated SNPs, with 12, seven and six candidate genes related to ASI, FF, and FM traits, respectively. Notably, four SNPs were located close to the same candidate genes, resulting in 22 unique candidate genes being identified in the present analysis.

**Figure 5:**
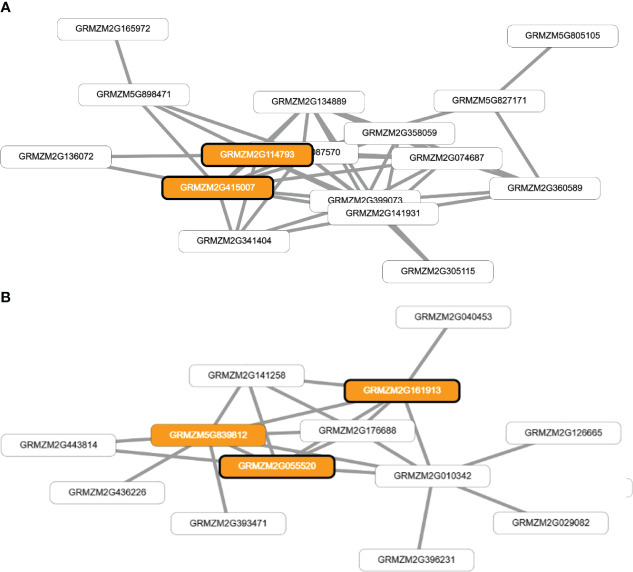
Visual representation of co-functional networks for flowering time traits in tropical maize. Panel **(A)** shows the network for anthesis-silking interval (ASI) and female flowering (FF) traits, while panel **(B)** displays the network for male flowering (FM). The networks were constructed using candidate genes identified through genome-wide association studies and prioritized using MaizeNet, a database of maize functional genomics. White boxes denote all the genes in the network, while orange boxes highlight genes that are associated with biological processes related to flowering time and reproduction, as identified by gene ontology (GO) annotations. The orange boxes with bold borders indicate genes identified by GWAS or through the prioritization analysis in MaizeNet.

The application of network-assisted prioritization using the MaizeNet database revealed 93 additional candidate genes associated with flowering time and reproductive processes. These genes were found to be involved in biological processes related to ASI (20 genes), FF (19 genes), and FM (54 genes) (**Supplementary Table** **S5**). The analysis also identified two co-functional networks that were found to be significantly enriched for genes related to single-organism reproductive behavior and the regulation of flower and reproductive development (p<0.0005). These networks identified four genes that were directly associated with the traits of FF and ASI (GRMZM2G114793 and GRMZM2G415007), and FM (GRMZM2G055520 and GRMZM2G161913) as shown in **Figure 5**.

The genes GRMZM2G114793 (bip1 - Binding protein homolog 1) and GRMZM2G415007 (bip2 - Binding protein homolog 2) were found to have orthologs in *Arabidopsis thaliana*, which encode BINDING PROTEIN 3.). The genes GRMZM2G055520 and GRMZM2G161913 have orthologs in *Arabidopsis thaliana* that encode EARLY FLOWERING 7 and EARLY FLOWERING 8, respectively. These genes are known to play a role in the control of flowering time in plants. Additionally, these four genes (GRMZM2G114793, GRMZM2G415007, GRMZM2G055520, and GRMZM2G161913) have an ontology associated with the stage of anthesis, or the beginning of flowering, in various cereal plants, including the silking stage in maize and the whole plant flowering stage.

### 3.4 Genomic prediction

The performance of four approaches (UTUE, UTME, MTUE, and MTME) for predicting flowering traits in tropical maize were compared using Bayesian and deep learning models. For this purpose, the approaches were evaluated in two scenarios: 1) selection of random training and validation datasets in each environment, and 2) prediction of Iguatemi season 2 using other environments as training datasets (Iguatemi season 1, DT1, Cambira and Sabaudia: DT2, and Cambira and Sabaudia + Iguatemi season 1: DT3).

#### 3.4.1 Selection randomly into independent training and validation datasets in each environment (scenario I)

*Predicting accuracy for uni-trait and multi-trait approaches in a single-environment (UTUE and MTUE)*:

The results of the study indicate that the multi-trait approach leads to higher prediction accuracy for all traits in each of the four environments evaluated, compared to the uni-trait approach. According to **Table 2**, prediction accuracies ranged from 0.11 to 0.73 for the uni-trait approach and from 0.16 to 0.73 for the multi-trait approach. The multi-trait approach, using the deep learning model, yielded the highest prediction accuracy (**Table 2**). On average, multi-trait genomic selection models (MCMCglmm and deep learning) had higher (not significantly) prediction accuracy than uni-trait genomic selection models. Particularly, the largest improvement in prediction accuracy (26.6%) was observed when using the multi-trait approach with the MCMCglmm model, while the smallest improvement (2.2%) was observed when using the deep learning model. However, the highest prediction accuracy was obtained using the deep learning model for all traits in each of the environments, when UTUE and MTUE approaches were considered (**Table 2**). This suggests that the deep learning model is less sensitive to the use of uni- or multi-trait approaches. The highest (not significantly) prediction accuracies were obtained for the Cambira environment, while the lowest (not significantly) was obtained for the Iguatemi Season 2 environment, for all traits in both uni- and multi-trait approaches, and for both the MCMCglmm and deep learning models.

**Table 2:** Predictive ability estimates for flowering time traits in a tropical maize panel across four environments (Cambira, Sabaudia, Iguatemi season 1, and Iguatemi season 2) using four different approaches: Uni-Trait-Uni-Environment (UTUE), Uni-Trait-Multi-Environment (UTME), Multi-Trait-Uni-Environment (MTUE), and Multi-Trait-Multi-Environment (MTME).

Environment	Trait	UTUE		UTME		MTUE		MTME	
		MCMCglmm	DL	BGGE	DL	MCMCglmm	DL	BMTME	DL
Cambira	FF	0.39e	0.62a	0.61ab	0.63a	0.45d	0.61ab	0.58c	0.59bc
	MF	0.42e	0.73ab	0.74a	0.73ab	0.49d	0.73bc	0.72bc	0.70c
	ASI	0.42bcd	0.5a	0.41d	0.44b	0.44bc	0.51a	0.41d	0.42cd
Sabaudia	FF	0.43c	0.49a	0.47ab	0.47ab	0.46abc	0.47ab	0.44bc	0.44abc
	MF	0.38e	0.58a	0.55abc	0.58ab	0.54bc	0.57abc	0.50d	0.56bc
	ASI	0.26c	0.31ab	0.30bc	0.30ab	0.29bc	0.32ab	0.28bc	0.33a
Iguatemi Season1	FF	0.36c	0.46ab	0.48a	0.46ab	0.45b	0.47a	0.46ab	0.42b
	MF	0.33d	0.55a	0.54a	0.51ab	0.45bc	0.54a	0.44c	0.48b
	ASI	0.12b	0.15ab	0.14ab	0.14ab	0.16ab	0.18a	0.13ab	0.13ab
Iguatemi Season2	FF	0.31d	0.42ab	0.43b	0.43b	0.42b	0.46a	0.33d	0.37c
	MF	0.29b	0.43a	0.31b	0.31b	0.29b	0.43a	0.31b	0.33b
	ASI	0.11c	0.27a	0.27a	0.25a	0.19b	0.27a	0.25a	0.24a

MCMCglmm, MCMC Generalised Linear Mixed Model; DL, Deep Learning; BGGE, Bayesian Genomic Genotype × Environment Interaction; BMTME, Bayesian Multi-Trait Multi-Environment. Statistical significance between different models is noted by lowercase letters. Different letters show the statistical significance at p< 0.01 according to the Tukey–Kramer test. The estimates are based on an average of 50 cross-validation cycles.

*Predicting accuracies for uni-trait and multi-trait approaches in multi-environments (UTME and MTME)*:

In contrast to the analysis of a single environment, the prediction accuracy of the uni-trait model was found to be higher than that of the multi-trait model in most cases, as shown in **Table 2**. The prediction accuracies ranged from 0.14 (for ASI, in Iguatemi season 1, using BGGE and deep learning models) to 0.74 (for MF, in Cambira, using the BGGE model) and from 0.13 (for ASI, in Iguatemi season 1, using BMTME and deep learning models) to 0.72 (for MF, in Cambira, using the BMTME model) for the uni-trait and multi-trait approaches, respectively. Regardless of the GS model used, the multi-trait analysis showed lower (not significantly) prediction accuracies than the single-trait model, except in ASI Cambira (BMTME), ASI Sabaudia (DL) and MF Iguatemi season 2 (BMTME and DL). On average, the GS models in the single-trait analysis had 8.3% (BGGE-UTME over BMTME-MTME) and 4.5% (DL-UTME over DL-MTME) higher (not significantly) prediction accuracies than GS models in the multi-trait analysis. Similarly, in the single environment analysis, deep learning models had the highest prediction accuracy on average. Overall, the results indicate that the Uni-Trait-Multi-Environment and Multi-Trait-Uni-Environment approaches are more efficient for predicting flowering traits. Furthermore, deep learning consistently emerged as the most accurate model across all approaches, demonstrating superior performance across various traits and environments.

#### 3.4.2 Prediction of flowering traits in Iguatemi season 2 (scenario II)

The prediction accuracy for scenario II was found to be generally higher than that of scenario I, as shown in **Tables 2**, **3**. **Table 3** presents the prediction accuracies for all flowering traits in the Iguatemi season 2 when the model was trained using three different datasets (DT1, DT2 and DT3) and four different approaches (Uni-Trait-Uni-Environment, Multi-Trait-Uni-Environment, Uni-Trait-Multi-Environment, and Multi-Trait-Multi-Environment). The prediction accuracies ranged from 0.20 to 0.66, 0.29 to 0.60, 0.24 to 0.68, and 0.13 to 0.60, respectively, for the four different approaches. It was found that the use of only season 1 of Iguatemi (DT1) performed the best among all scenarios. Additionally, the use of deep learning models was found to be more efficient (not significantly) for predicting flowering traits in tropical maize, with an improvement of 26.8% and 10.8% in the Uni-Trait and Multi-Trait approaches, respectively, when using DT1, and 2.5% and 1.6% in the Uni-Trait and Multi-Trait approaches, respectively, when using DT3.

**Table 3:** Estimates of predictive ability for flowering time traits in a tropical maize panel for the second season of Iguatemi (validation dataset).

Scenario	Trait	UTUE		UTME		MTUE		MTME	
		MCMCglmm	DL	BGGE	DL	MCMCglmm	DL	BMTME	DL
DT1	FF	0.53	0.66	–	–	0.67	0.68	–	–
	MF	0.44	0.51	–	–	0.49	0.54	–	–
	ASI	0.20	0.28	–	–	0.24	0.29	–	–
DT2	FF	–	–	0.58	0.59	–	–	0.31	0.59
	MF	–	–	0.41	0.41	–	–	0.13	0.40
	ASI	–	–	0.31	0.29	–	–	0.20	0.34
DT3	FF	–	–	0.59	0.60	–	–	0.58	0.60
	MF	–	–	0.41	0.42	–	–	0.43	0.41
	ASI	–	–	0.31	0.32	–	–	0.34	0.36

MCMCglmm, MCMC Generalized Linear Mixed Model; DL, Deep Learning; BGGE, Bayesian Genomic Genotype × Environment Interaction; BMTME: Bayesian Multi-Trait Multi-Environment. DT1 (Iguatemi season 1), DT2 (Cambira and Sabaudia) and DT3 (Cambira, Sabaudia and the first season of Iguatemi) represent the training dataset. Four different approaches were used: Uni-Trait-Uni-Environment (UTUE), Uni-Trait-Multi-Environment (UTME), Multi-Trait-Uni-Environment (MTUE), and Multi-Trait-Multi-Environment (MTME) - indicates that the model was not run in this approach and scenario.

## 4 Discussion

### 4.1 Genetic determinants of flowering traits in tropical maize

The flowering traits of crops are crucial for yield and seed quality (Helal et al., 2021). In this study, 31 significant SNP loci were identified that regulate flowering traits across two consecutive seasons. Of these, approximately 50% of significant SNPs were located on chromosomes 1, 2, and 3, which is consistent with previous research that found over 33% of loci associated with flowering on these chromosomes (Li et al., 2016; Liu et al., 2019; Maldonado et al., 2019). Additionally, 9 SNPs (82%) associated with ASI were found on chromosomes 1, 3, and 4; for FF trait, 70% (7/10) of SNPs were found on chromosomes 2, 7, and 8; and for FM trait, 4 SNPs (44%) were distributed on chromosomes 1 and 7 (**Supplementary Table** **S3**). Previous studies have also identified significant SNPs associated with flowering traits in maize on similar chromosomes (Li et al., 2016; Liu et al., 2019; Maldonado et al., 2019; Shi et al., 2022), suggesting that these regions may contain genes that play a critical role in controlling flowering time variation in maize. The phenotypic variation explained by significant SNPs in this study ranged from 6.7 to 9.6% and was evenly distributed among traits, indicating that many significant SNPs of small effects contribute to genetic variation in flowering time in maize (Maldonado et al., 2019).

The study identified two potential pleiotropic loci that had an impact on both female and male flowering traits. The use of multivariate Bayesian regression (as suggested by Maldonado et al., 2019) allowed for the detection of pleiotropic genetic variants that are correlated with multiple traits by analyzing the Bayes factor and PPA. The PPA values of 0.99 and 0.74 for SNPs S6_150165479 and S5_217372319, respectively, provided strong evidence of the simultaneous association of these two loci with both FF and FM traits. Additionally, the high values of log10 (BF) (> 5.1) were considered to be strong evidence against the null hypothesis of no association and were higher than those found in previous association studies (Legarra et al., 2018). The correlation analysis results also showed a high and significant correlation between FF and FM, which supports the idea that FF and FM share similar loci. The study also found similarities to previous research by Li et al. (2016) who identified two pleiotropic significant SNPs located in the same bin (6.05) of loci S6_150165479, indicating that this region affects both female and male flowering time. These discoveries of pleiotropic significant SNPs could aid in understanding the molecular mechanisms of flowering time in maize.

GWAS is a powerful tool for identifying genetic variants associated with specific traits in maize. Studies such as those by Xiao et al. (2016); Coan et al. (2018); Maldonado et al. (2019), and Shi et al. (2022) have used GWAS to identify key genetic variants underlying phenotypic variation in several maize traits. Additionally, Wallace et al. (2014) found that the majority of the variance in maize can be explained by within-gene and gene-proximal SNPs (at about 1–5 kb). By using high-resolution GWAS, it may be possible to identify loci that significantly affect maize flowering time within candidate genes or in proximity to them. Therefore, GWAS approaches can be a useful tool for understanding the genetic basis of flowering time in maize and for identifying potential targets for crop improvement.

The association analysis identified several markers associated with flowering traits in maize, which explain up to 9.6% of phenotypic variation individually, and between 67 and 86% of the trait phenotypic variation considering all significant markers. This result is consistent with previous studies on traits related to flowering time in maize (Salvi et al., 2009; Liu et al., 2019; Maldonado et al., 2019).Moreover, it is worth noting that the LD pattern exhibits a rapid decline within a 2.7 kb range, which aligns with the findings reported by Coan et al. (2018) and Maldonado et al. (2019). This LD pattern indicated that candidate genes should be located within a 2.7 kb region upstream and downstream of significant SNPs. The gene-prioritization and co-functional network approach found that four genes were significantly associated with the stage at flowers open, anthesis and silking in some cereal plants such as maize. In this regard, hundreds of genes in plants have been extensively studied in *Arabidopsis*. In this study, ortholog genes for BINDING PROTEIN 3 (which control pollen germination and pollen tube elongation; Sato and Maeshima, 2019), orthologs associated with the stage of anthesis or the beginning of flowering (particularly important in the sporophyte reproductive stage; Xiang et al., 2011), and EARLY FLOWERING genes (which play a crucial role in determining when a plant flower; Li et al., 2016; Li et al., 2019) have been identified. Particularly, two orthologs of EARLY FLOWERING genes, ELF7 and ELF8, were identified as candidate genes controlling flowering time in maize using gene-prioritization and subnetwork analysis of the MaizeNet database (Lee et al., 2019). These genes have been shown to cause rapid flowering in various situations where flowering would otherwise be delayed (He et al., 2004; Li et al., 2016). Additionally, ELF7 and ELF8 are known to regulate the expression of genes in the FLOWERING LOCUS C clade, which includes repressors such as MAF2 and FLM that play a role in multiple flowering pathways (He et al., 2004). The SNPs and candidate genes associated with flowering time phenotypes identified in this study can be integrated into molecular marker-assisted breeding programs and provide valuable genetic resources for future maize breeding efforts.

### 4.2 Multi-trait and multi-environment genomic prediction for flowering traits in maize

Genomic selection is a powerful strategy that has been proven to significantly improve the efficiency of breeding programs by increasing genetic gain and reducing selection time (Bhat et al., 2016). The goal of GS is to construct accurate prediction models using training populations that consist of individuals with both genotypic and phenotypic data. In practice, plant breeders often collect data for multiple traits in different environments and over multiple years. Studies have shown that prediction approaches based on Multi-Trait and Multi-Environments (MT-ME) are more accurate than Uni-Trait and Uni-Environment (UT-UE) approaches because they allow for the prediction of multiple traits simultaneously, which reduces the number of locations needed for subsequent selection trials (Tolhurst et al., 2019; Larkin et al., 2021; Sandhu et al., 2022). Despite the benefits of using MT-ME approaches, few GS studies have adopted them due to the complexity of the models (Cuevas et al., 2017). Therefore, in this study, different models based on MT-ME approaches were evaluated and compared with UT-UE approaches to predict flowering traits in inbred lines of tropical maize.

In the scenario I, when considering selection randomly into independent training and validation datasets in each environment, the multi-trait approach performed 14.4% superior to the Uni-Trait approach for the Uni-environment, while in the Multi-Environment approach, the Uni-Trait approach performed 6.4% superior to the Multi-Trait approach. Notably, regardless of the approach, the Deep Learning model showed a higher prediction accuracy (**Table 2**). Additionally, the Deep Learning model was significantly superior to the MCMCglmm (Uni-Trait-Uni-Environment and Multi-Trait-Uni-Environment) and BMTME (Multi-Trait-Multi-Environment) models. These results may be due to the ability of the Deep Learning model to automatically capture complex interactions in its hidden layers without the need to specify the covariates corresponding to interactions between traits or environments in the predictor, as previously noted by Montesinos-López et al. (2018). It is worth noting that similar results have been observed by Montesinos-López et al. (2018) where the Deep Learning model performed superiorly to other models when the genotype-environment interaction (Uni-Environment) is not considered, but its advantages diminished when the genotype-environment component is included in the model, which is consistent with the findings of this study.

In scenario II, when predicting the second season of Iguatemi, utilizing information from the first season of Iguatemi (DT1) was found to be more accurate than utilizing information from other environments (DT2 and DT3). This may be due to the high correlation observed between the first and second seasons of Iguatemi for traits such as FF (r = 0.68), MF (r = 0.53), and anthesis-silking interval (ASI: r = 0.30), compared to the correlation between these traits in other environments. Furthermore, the results of this scenario differed from those of scenario I, as the prediction accuracy for the FF trait was found to be superior to that of the MF trait. This may be due to the high correlation observed among all environments and traits for the FF trait (as shown in **Figures 2, 3**), as previously reported by Sandhu et al. (2022) and Montesinos-López et al. (2016), who mention that a high correlation between traits improves prediction accuracies and highlights the importance of using multi-trait models. Additionally, for the ASI trait, which has a low correlation among traits and environments, as well as a low heritability (h^2 = ^0.29), the Deep Learning model in the DT3 (Multi-Trait-Multi-Environment) approach was found to be more effective than models in the DT1 (Uni-Trait-Uni-Environment and Multi-Trait-Uni-Environment) approach. This suggests that Multi-Trait-Multi-Environment approaches may be useful for increasing predictions for primary traits with low heritability when a secondary trait is highly correlated and has high heritability (as reported by Sandhu et al., 2022). As noted by Cui et al. (2020), heritability can vary depending on the genetic architecture of traits, with traits such as flowering date being controlled by several major genetic loci that have high heritabilities. This study found that the flowering traits had moderate to high heritabilities (FF: 0.72, MF: 0.66, and ASI: 0.29) (as reported by Cui et al., 2020; Maldonado et al., 2020). As expected, the prediction accuracy was moderate to high (as reported by Zhang et al., 2017), with higher prediction accuracies observed for traits with higher heritability compared to those with lower heritability. Similar results have been observed in previous studies, with high positive correlations between heritability and prediction accuracy values (as reported by Nyine et al., 2017; Cui et al., 2020; Kaler et al., 2022). Notably, the Deep Learning model showed higher prediction accuracy compared to other models, regardless of the heritability of the trait. This is in line with the findings of Alves et al. (2020) who found that artificial neural network models had a higher prediction accuracy compared with GBLUP for traits with moderate heritability, indicating that neural network models may be a promising alternative tool for genomic prediction, independent of the contribution of genetic effects (as reported by Maldonado et al., 2020).

In all scenarios, the use of Deep Learning models resulted in higher prediction accuracy compared to other models for all traits (except BGGE in UTME, since it had similar predictions). This suggests that the Deep Learning model is less sensitive to random variations among seasons and correlations between traits and that it does not require the consideration of “genotype x environment” interactions and prior information on the covariance matrices of traits (genetic and residual) for training and constructing the predictive model (Montesinos-López et al., 2018). In this regard, Maldonado et al. (2020) highlighted that machine learning-based GP models can treat response variables as an implicit function of input variables (e.g., environmental components) through non-linear and highly complex functions, which implies that these models can effectively increase prediction accuracy without the need to pre-specify interaction terms.

In this study, it was shown that Deep Learning models based on Uni- or Multi-Trait and Uni- or Multi-Environment approaches outperformed Bayesian Genomic Selection models (MCMCglmm and BMTME). It should be noted that BGGE achieved the same level of prediction accuracy as DL in UTME, however, the computational time required for BGGE was approximately three times longer than that of DL (data not shown). Similar results were observed by Maldonado et al. (2020), which indicated that DL models require significantly less computational time (approximately 16 times less) compared to traditional Bayesian models. The superiority of Deep Learning models in GS over traditional mixed model-based approaches has been previously reported in the literature by Sandhu et al. (2021), Zingaretti et al. (2020), Montesinos-López et al. (2018), and Maldonado et al. (2020). According to Sandhu et al. (2022), Deep Learning models are highly flexible in understanding the complex interactions present in datasets, and they can infer trends present in datasets better than traditional models. The results of this study confirm the importance of Deep Learning models for increasing prediction accuracy in GS, which holds promise for accelerating crop breeding progress.

## 5 Conclusion

In conclusion, this study highlights the effectiveness of deep learning models in genomic selection studies for predicting complex flowering-related traits in tropical maize. Deep learning models outperformed other models (except for BGGE in UTME where similar predictions were observed) indicating their superior accuracy across all traits and scenarios. This suggests that Multi-Traits deep learning models are less affected by low or negative correlations among traits. Moreover, these models have the advantage of learning patterns directly from the data without relying on prior assumptions, making them an attractive alternative to traditional Multi-Trait and Multi-Environment based models. Among the deep learning models, the MTUE model consistently demonstrated the highest prediction accuracies on average. Therefore, it is recommended to use this model in breeding programs, especially for predicting traits that are challenging or expensive to phenotype, or those with low levels of correlation. Additionally, deep learning models should be incorporated into the toolkit of plant breeders to accelerate crop breeding progress and improve genetic gain for quantitative traits. On the other hand, this study identified several loci in genomic regions associated with flowering time in tropical maize, which have variable contributions to phenotypic expression. These findings can be utilized in marker-assisted selection programs, where the loci identified can be target to improve breeding outcomes. Additionally, through the co-functional network approach (post-GWAS), orthologs of EARLY FLOWERING genes were identified, which offer potential targets for genome editing programs focused on improving flowering traits. These discoveries provide valuable insights into the genetic architecture and underlying mechanisms of flowering-related traits in tropical maize, which can be incorporated into breeding programs for further advancements.

## Data availability statement

The original contributions presented in the study are included in the article/**Supplementary Material**. Further inquiries can be directed to the corresponding author.

## Author contributions

FM-P, CM, and CS conceived the research plans. LH, CAM, and RU performed the data curation. CM and FM-P analyzed the genomic data and wrote the first draft of the manuscript. CAM, LH, and CS supervised the field experiments. FM-P, CAM, RU, and LH reviewed and edited the final version of manuscript. All authors reviewed and approved the paper for publication. All authors contributed to the article and approved the submitted version.
