# Supplementary material for: Multi-trait and multi-environment genomic prediction for flowering traits in maize: a deep learning approach
Source: Front Plant Sci. 2023 Aug 1;14:1153040. doi: 10.3389/fpls.2023.1153040 (PMC10428628; doi:10.3389/fpls.2023.1153040)
Supplement: Supplementary file 1 [file Table_1.docx]

Supplementary Material

Multi-Trait and Multi-Environment Genomic Prediction for Flowering traits in Maize: A Deep Learning Approach

Freddy Mora-Poblete; Carlos Maldonado*; Luma Henrique; Renan Uhdre; Carlos Alberto Scapim; Claudete Aparecida Mangolim

*** Correspondence:** Carlos Maldonado: [cmaldo1782@gmail.com](mailto:cmaldo1782@gmail.com)

# Supplementary Table S1: Description of the genetic backgrounds collected of each inbred line.

| Genotype | Kernel | Genotype | Kernel |
| --- | --- | --- | --- |
| 100-F94 | Field Corn | 254-P1780 | Popcorn |
| 101-F95 | Field Corn | 255-P18 | Popcorn |
| 102-FLASH21.H11.1-46 | Field Corn | 256-P19 | Popcorn |
| 106-HTMV16.4.3 | Field Corn | 257-P20 | Popcorn |
| 107-HTMV17.1.3 | Field Corn | 258-P3.1.2 | Popcorn |
| 108-HTMV18.5.2 | Field Corn | 259-P3.3T | Popcorn |
| 109-IAC12501-2 | Popcorn | 25-AL-PIRATININGA23.1.2 | Field Corn |
| 10-AG105110.1.1 | Field Corn | 260-P4(GP4) | Popcorn |
| 110-IAC12502-2 | Popcorn | 261-P4.4 | Popcorn |
| 111-IAC12504-2 | Popcorn | 262-P6.1 | Popcorn |
| 117-P11 | Popcorn | 263-P6.11 | Popcorn |
| 118-P12 | Popcorn | 264-P7.2.1 | Popcorn |
| 119-P12.2 | Popcorn | 265-P7.2.3 | Popcorn |
| 11-AG105113.1.1 | Field Corn | 266-P7.2.4 | Popcorn |
| 120-P14 | Popcorn | 267-P7.4.11 | Popcorn |
| 121-P14.1 | Popcorn | 268-P7.4.5 | Popcorn |
| 122-P16 | Popcorn | 269-P7.17.1 | Popcorn |
| 123-P3 | Popcorn | 26-AL-PIRATININGA8.4.1 | Field Corn |
| 124-P5 | Popcorn | 270-P8.1.1 | Popcorn |
| 125-P5.1 | Popcorn | 271-P8.1.5.10 | Popcorn |
| 126-P8.1.1.5 | Popcorn | 272-P8.1.5.13 | Popcorn |
| 129-P9.10.1 | Popcorn | 273-P8.1.5.5 | Popcorn |
| 12-AG105130.1.1 | Field Corn | 274-P8.1.5.9 | Popcorn |
| 1-30.126 | SweetCorn | 275-P8.2 | Popcorn |
| 131-P9.4.2 | Popcorn | 276-P8.2MULT | Popcorn |
| 132-P9.5.2 | Popcorn | 277-P8.2.2.2 | Popcorn |
| 135-P9.7.3 | Popcorn | 279-P8.2.2.5 | Popcorn |
| 136-P9.8.2 | Popcorn | 27-AM400112.1.3 | Field Corn |
| 13-AG10517.5.1 | Field Corn | 280-P9.1 | Popcorn |
| 140-PA091355-2 | Popcorn | 281-P9.1.2 | Popcorn |
| 141-PA170ROXO315-2 | Popcorn | 282-P9.1.3 | Popcorn |
| 142-PA170ROXO323-2 | Popcorn | 283-P9.1.6 | Popcorn |
| 143-PARA17278-2 | Popcorn | 284-P9.11.1 | Popcorn |
| 144-POP10276.1 | Field Corn | 285-P9.12.1 | Popcorn |
| 145-PR023242-2 | Popcorn | 287-P9.3.2 | Popcorn |
| 149-SAM260-2 | Popcorn | 288-P9.4.5 | Popcorn |
| 14-AG405110.3.1 | Field Corn | 28-AM400114.1.2 | Field Corn |
| 150-SAM262-2 | Popcorn | 290-P9.5.1 | Popcorn |
| 152-SAM266-2 | Popcorn | 291-P9.7.2 | Popcorn |
| 153-SD02 | SweetCorn | 292-P9.8.1 | Popcorn |
| 154-SD03 | SweetCorn | 294-POP101195.2 | Field Corn |
| 155-SD08 | SweetCorn | 295-POP101197-1 | Field Corn |
| 156-SD11 | SweetCorn | 296-POP101201.3 | Field Corn |
| 157-SD12 | SweetCorn | 297-POP102166.5 | Field Corn |
| 158-SD13 | SweetCorn | 298-POP10290.1 | Field Corn |
| 15-AG405110.3.2 | Field Corn | 299-POP10291.2 | Field Corn |
| 162-TORK54.H20.3-146 | Field Corn | 29-AM40014.3.2 | Field Corn |
| 164-URUG29897-2 | Popcorn | 300-POP10380.5 | Field Corn |
| 165-URUG29898-2 | Popcorn | 302-POP10388.1 | Field Corn |
| 166-VICOSAL59 | Popcorn | 303-POP201192.1 | Field Corn |
| 167-P16.12 | Popcorn | 306-POP202177.1 | Field Corn |
| 169-BRS10305-11-1-1 | Field Corn | 307-POP20276.1 | Field Corn |
| 16-AG40517.1.2 | Field Corn | 308-POP20288.2 | Field Corn |
| 173-29-14 | Field Corn | 309-POP20351.2 | Field Corn |
| 174-29-154 | Field Corn | 30-AM40016.2.1 | Field Corn |
| 175-30.11 | SweetCorn | 310-POP20356.1 | Field Corn |
| 176-30.23 | SweetCorn | 311-PR023245-1 | Popcorn |
| 177-30.29 | SweetCorn | 312-PREMIUM28.H13.2-67 | Field Corn |
| 178-30F3369.H26.1-188 | Field Corn | 313-PREMIUM29.H13.3 | Field Corn |
| 17-AG40517.1.3 | Field Corn | 315-SAM274-2 | Popcorn |
| 180-30F3371.H26.2-194 | Field Corn | 317-SPEED81.H33.1-230 | Field Corn |
| 182-30F9875.H29.2-210 | Field Corn | 318-STRIKE67.H25.1-182 | Field Corn |
| 183-31-88 | Field Corn | 319-TORK53.H20.2-143 | Field Corn |
| 184-31-124 | Field Corn | 31-AM40019.1.3 | Field Corn |
| 185-31-33 | Field Corn | 320-TORK54.H20.3-145 | Field Corn |
| 186-A1154527.H16.1-73 | Field Corn | 321-TORK55.H20.3-149 | Field Corn |
| 187-A256062.H23.2-167 | Field Corn | 323-VICOLAL77 | Popcorn |
| 188-A256063.H23.2-170 | Field Corn | 324-W57 | Field Corn |
| 18-AG40519.2.1 | Field Corn | 327-P9.5.3 | Popcorn |
| 191-AG80807.H3.1-3 | Field Corn | 32-AM400210.1.1 | Field Corn |
| 192-AG80808.H3.2-6 | Field Corn | 3-30.272 | SweetCorn |
| 193-ANGELAL66 | Popcorn | 33-AM40026.1.3 | Field Corn |
| 194-ANGELAL70 | Popcorn | 34-AM40025.2.3 | Field Corn |
| 195-ANGELAL71 | Popcorn | 35-AM40027.2.1 | Field Corn |
| 197-AVANT10.H5.1-12 | Field Corn | 36-AM40028.1.2 | Field Corn |
| 198-AVANT12.H5.3 | Field Corn | 37-AM60617.1.7 | Field Corn |
| 199-AVANT13.H5.4-21 | Field Corn | 38-AM60625.1.2 | Field Corn |
| 19-AG601823H12.1-55 | Field Corn | 39-AM60635.2.2 | Field Corn |
| 200-AVANT14.3H5-24 | Field Corn | 40-AM60642.2.1 | Field Corn |
| 202-BEIJA-FLORL55 | Popcorn | 41-AM6065.2.1 | Field Corn |
| 203-BEIJA-FLORL76 | Popcorn | 42-AM81116.1.3 | Field Corn |
| 205-CD30389.H4.2-258 | Field Corn | 43-AM81116.2.3 | Field Corn |
| 208-CHZM1313466-2 | Popcorn | 44-AM8112.2.2 | Field Corn |
| 209-CML12 | Field Corn | 45-AM8112.2.4 | Field Corn |
| 20-AG601826H12.2-61 | Field Corn | 46-AM81148.1.2 | Field Corn |
| 210-CML13 | Field Corn | 47-AM9972.4.1 | Field Corn |
| 211-CML19 | Field Corn | 48-AM9976.3.1 | Field Corn |
| 212-CML22 | Field Corn | 49-AM9977.1.2 | Field Corn |
| 213-DAS2C59995.H34.4-276 | Field Corn | 50-AM9978.1.2 | Field Corn |
| 214-DAS42279.H31.1-22 | Field Corn | 51-AM9979.3.1 | Field Corn |
| 215-DKB35019.H9.1-43 | Field Corn | 54-ARMZ13050153-2 | Popcorn |
| 216-DKB35076.H30.1-213 | Field Corn | 57-BARAOVICOSA126-2 | Popcorn |
| 217-DKB35077.H30.1-216 | Field Corn | 58-BARAOVICOSA130-2 | Popcorn |
| 218-DKB35078.H30.1-219 | Field Corn | 59-BEIJA-FLORL51 | Popcorn |
| 219-DKB44073.H28.1-240 | Field Corn | 61-BOYA46211-2 | Popcorn |
| 21-AG909058.H21.2-158 | Field Corn | 62-CD30388.H4.1-225 | Field Corn |
| 221-DKB74736.H17.2-82 | Field Corn | 6-30F3370H26.1 | Field Corn |
| 222-P8.1.5.4 | Popcorn | 63-CD31617.6.3 | Field Corn |
| 223-DKB74737.H17.2-89 | Field Corn | 64-CD31619.2.1 | Field Corn |
| 224-DKB74738.H17.2-92 | Field Corn | 65-CD31624.1.3 | Field Corn |
| 225-DKB74740.H17.3-93 | Field Corn | 66-CD3167.2.2 | Field Corn |
| 226-DKB74741.H17.3-101 | Field Corn | 67-CD3169..3.3 | Field Corn |
| 227-DKB74743.H17.4-107 | Field Corn | 68-CD39313.1.1 | Field Corn |
| 228-DKB74744.H17.4-110 | Field Corn | 70-CD39326.1.3 | Field Corn |
| 229-DKB74745.H17.5-115 | Field Corn | 71-CD3935.1.3 | Field Corn |
| 22-AG909058-188 | Field Corn | 72-CD3936.1.2 | Field Corn |
| 230-DKB74747.H17.5-121 | Field Corn | 73-DAS2C59596.H36.5-279 | Field Corn |
| 231-DKB74748.H17.5-124 | Field Corn | 74-DAS2C59992.H34.1-267 | Field Corn |
| 232-DKB74750.H17.6-130 | Field Corn | 7-53F | Field Corn |
| 233-FLASH20.H11.1-46 | Field Corn | 75-DAS2C59993.H34.2-270 | Field Corn |
| 234-FLASH22H11.1-52 | Field Corn | 76-DAS2C59994.H34.3-273 | Field Corn |
| 235-FORT84.H6.1-239 | Field Corn | 78-DKB74442.H17.4-104 | Field Corn |
| 236-FORT85.H6.2-242 | Field Corn | 79-DKB74749.H176-127 | Field Corn |
| 237-FORT86.H6.3-245 | Field Corn | 81-DKB74735.H17.1-9 | Field Corn |
| 238-FORT87.H6.4-248 | Field Corn | 82-DKB74746.H17.5-118 | Field Corn |
| 239-GP1 | Popcorn | 83-DKB74749.H17.1 | Field Corn |
| 23-AL-PIRATININGA13.7.1 | Field Corn | 84-F102 | Field Corn |
| 240-GP12 | Popcorn | 87-F145 | Field Corn |
| 241-GP14 | Popcorn | 88-F15 | Field Corn |
| 244-P1.12 | Popcorn | 89-F154 | Field Corn |
| 245-P1.19 | Popcorn | 8-A256060H23.3-164 | Field Corn |
| 246-P1.3 | Popcorn | 90-F157 | Field Corn |
| 247-P1.8 | Popcorn | 91-F28 | Field Corn |
| 248-P10(GP10) | Popcorn | 92-F41 | Field Corn |
| 249-P11.1 | Popcorn | 93-F46 | Field Corn |
| 24-AL-PIRATININGA21.1.2 | Field Corn | 95-F55 | Field Corn |
| 250-P11.2 | Popcorn | 96-F77 | Field Corn |
| 252-P13(GP13) | Popcorn | 97-F80 | Field Corn |
| 253-P15 | Popcorn | 9-A256060H23.3-176 | Popcorn |

## Supplementary Table S2. Summary of information on single nucleotide polymorphism (SNP) markers and Linkage disequilibrium (LD) decay patterns in all chromosomes of the maize population studied.

| Chromosome | Number of SNPs | Position (pb) | | Linkage Disequilibrium |
| --- | --- | --- | --- | --- |
|  |  | First | Last |  |
| 1 | 39180 | 38222 | 275861066 | 2.67 |
| 2 | 37506 | 40724 | 244417305 | 2.52 |
| 3 | 34949 | 191169 | 235520333 | 2.13 |
| 4 | 27002 | 217040 | 246840261 | 5.35 |
| 5 | 35783 | 12711 | 223658670 | 2.34 |
| 6 | 23475 | 169964 | 173881702 | 3.25 |
| 7 | 25033 | 180204 | 182128999 | 2.11 |
| 8 | 25588 | 204228 | 181043617 | 3.50 |
| 9 | 22435 | 61292 | 159668042 | 2.65 |
| 10 | 20682 | 128669 | 150847940 | 2.57 |
| Total | 291633 | - | - | 2.70 |

## Supplementary Table S3. Putative pleiotropic loci associated with the phenotypic variation of female flowering (FF) and male flowering (MF) in tropical maize.

| SNP | Traits | Log10  (Bayes Factor) | Posterior  Odds | Posterior Probability  of Association |
| --- | --- | --- | --- | --- |
| S5_217372319 | FF-MF | 5.2 | 2.9 | 0.74 |
| S6_150165479 | FF-MF | 6.8 | 126.6 | 0.99 |

## Supplementary Table S4. Summary of candidate genes detected in an association study for the traits of Female/Male Flowering time (FF and MF, respectively) and Anthesis–Silking Interval (ASI) measured in inbred lines of tropical maize in two growing seasons (2020 and 2021).

| Trait | Iguatemi  Season | SNP | GeneID | Putative Function |
| --- | --- | --- | --- | --- |
| ASI | 1 | S1_12340947 | GRMZM2G000823 | ATP-dependent RNA helicase DBP5 |
|  | 1 |  | GRMZM2G075958 | Photosynthetic NDH subunit of subcomplex B 2 chloroplastic |
|  | 1 |  | GRMZM2G000823 | DEAD-box ATP-dependent RNA helicase 38 |
|  | 1 |  | GRMZM2G075958 | Photosynthetic NDH subunit of subcomplex B 2 chloroplastic |
|  | 1 | S2_142739572 | GRMZM2G146472 | Alba DNA/RNA-binding protein |
|  | 2 | S1_214720998 | GRMZM2G047238 | Stromal cell-derived factor 2 |
|  | 2 | S3_122398302 | GRMZM2G114613 | Protein LSD1 - 18S subunit ribosomal protein |
|  | 2 | S3_122398313 | GRMZM2G114613 | Protein LSD1 - 18S subunit ribosomal protein |
|  | 2 | S3_122398320 | GRMZM2G114613 | Protein LSD1 - 18S subunit ribosomal protein |
|  | 2 | S4_245029688 | GRMZM2G074423 | Plant-specific domain TIGR01589 family protein |
|  | 2 |  | GRMZM2G074414 | AP-3 complex subunit mu |
| FF | 1 | S10_14797601 | GRMZM2G021676 | Uncharacterized protein |
|  | 1 |  | GRMZM2G021706 | sweet13b - sugars will eventually be exported transporter13b - Bidirectional sugar transporter SWEET |
|  | 1 | S7_468747 | GRMZM2G152059 | Legume lectins beta domain containing protein |
|  | 1 | S8_143046924 | GRMZM2G165428 | Putative leucine-rich repeat protein kinase family protein |
|  | 2 | S2_15002111 | GRMZM2G314412 | RING-type E3 ubiquitin transferase |
|  | 2 | S2_43966599 | GRMZM2G430411 | CLE family OsCLE401 protein |
|  | 2 | S2_47411894 | GRMZM2G131321 | Signal peptidase complex subunit 2 |
|  | 2 | S5_42052202 | GRMZM2G350626 | p-loop containing nucleoside triphosphate hydrolase superfamily protein |
| FM | 1 | S7_13731608 | GRMZM2G150091 | Thioredoxin |
|  | 1 |  | GRMZM2G149916 | 2-methoxy-6-polyprenyl-14-benzoquinol methylase mitochondrial |
|  | 2 | S1_65858162 | GRMZM2G127150 | Cationic amino acid transporter |
|  | 2 | S2_222099831 | GRMZM2G075387 | Glycosyltransferase |
|  | 2 |  | GRMZM2G075431 | RNA pseudouridine synthase 6 chloroplastic |
|  | 2 | S7_8758861 | GRMZM2G312910 | Protein containing PDZ domain a K-box domain and a TPR region |
|  | 2 |  | GRMZM2G312839 | Galactose-1-phosphate uridyl transferase-like protein |

## Supplementary Table S5. Summary of candidate genes identified through an association study and prioritized by MaizeNet for their potential role in flowering-related traits in maize.

| Trait-Season | GeneID identified by GWAS in this study | GeneID identified by MaizeNet |
| --- | --- | --- |
| ASI-1 | GRMZM2G000823, GRMZM2G075958, GRMZM2G146472 | GRMZM2G380184, GRMZM2G032505, GRMZM2G002100, GRMZM2G455085, GRMZM2G171444, GRMZM2G155375 |
| ASI-2 | GRMZM2G047238, GRMZM2G114613, GRMZM2G074423, GRMZM2G074414, GRMZM2G074278 | GRMZM2G434910, GRMZM2G013936, GRMZM2G002765, GRMZM2G415007, GRMZM2G114793, GRMZM5G858887, GRMZM2G074317, GRMZM2G013908, GRMZM2G176029, GRMZM2G122805, GRMZM2G168953, GRMZM2G015132, GRMZM2G098569, GRMZM2G027209 |
| FF-1 | GRMZM2G021676, GRMZM2G152059, GRMZM2G165428 | GRMZM2G113373, GRMZM2G006578, GRMZM2G409893, GRMZM2G158679 |
| FF-2 | GRMZM2G314412, GRMZM2G430411, GRMZM2G131321, GRMZM2G350626 | GRMZM2G071071, GRMZM2G151549, GRMZM2G143588, GRMZM2G415007, GRMZM2G114793, GRMZM2G176029, GRMZM2G090528, GRMZM2G101571, GRMZM2G090669, GRMZM2G146951, GRMZM2G006117, GRMZM2G039811, GRMZM2G046005, GRMZM2G409104, GRMZM2G170281 |
| FM-1 | GRMZM2G150091 | GRMZM2G359314 |
| FM-2 | GRMZM2G127150, GRMZM2G075387, GRMZM2G075431, GRMZM2G312910, GRMZM2G312839 | GRMZM2G402708, GRMZM2G071304, GRMZM2G161913, GRMZM2G045704, GRMZM2G140033, GRMZM2G067789, GRMZM2G007810, GRMZM2G021864, GRMZM2G104920, GRMZM2G055520, GRMZM2G092433, GRMZM2G027867, GRMZM2G076075, GRMZM2G126742, GRMZM2G092451, GRMZM2G434910, GRMZM2G057159, GRMZM2G100794, GRMZM2G138410, AC208897.3_FG004, GRMZM2G117146, GRMZM2G052336, GRMZM2G001296, GRMZM2G129913, GRMZM2G123633, GRMZM2G115564, GRMZM2G051984, GRMZM2G058584, GRMZM2G133359, GRMZM5G891373, GRMZM2G036609, GRMZM2G083716, AC215201.3_FG005, GRMZM2G382914, GRMZM2G089136, GRMZM2G146951, GRMZM2G006117, GRMZM2G039811, GRMZM2G046005, GRMZM2G409104, GRMZM2G170281, GRMZM2G002220, GRMZM2G035417, GRMZM2G098545, GRMZM2G370915, GRMZM2G082385, GRMZM2G174481, GRMZM2G061620, GRMZM2G143462, AC217975.3_FG001, GRMZM2G046284, GRMZM2G012397, GRMZM2G057186 |

**
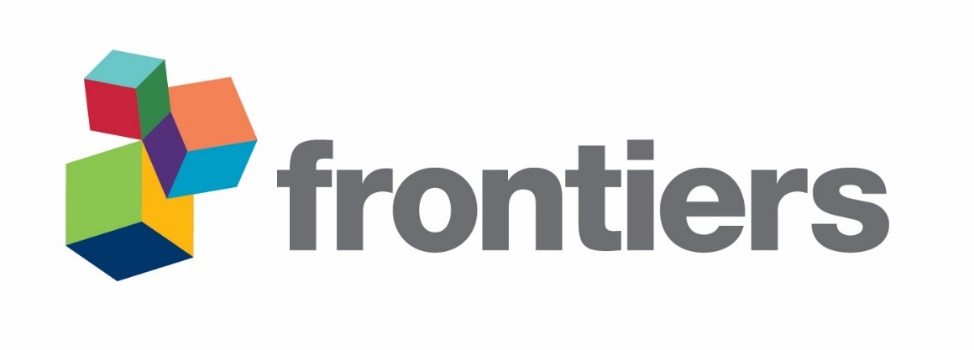
**
